# A Simple Colorimetric Assay for Sensitive Cu^2+^ Detection Based on the Glutathione-Mediated Etching of MnO_2_ Nanosheets

**DOI:** 10.3389/fchem.2021.812503

**Published:** 2021-12-24

**Authors:** Shurong Tang, Qiao Liu, Jie Hu, Wei Chen, Fengping An, Hui Xu, Hongbo Song, Yi-Wei Wang

**Affiliations:** ^1^ Faculty of Pharmacy, Fujian Medical University, Fuzhou, China; ^2^ College of Food Science, Fujian Agriculture and Forestry University, Fuzhou, China; ^3^ Wuyi University, Wuyishan, China

**Keywords:** colorimetric system, MnO_2_ mimetic enzyme, copper ions, rapid detection, water sample

## Abstract

In this paper, we developed a quick, economical and sensitive colorimetric strategy for copper ions (Cu^2+^) quantification via the redox response of MnO_2_ nanosheets with glutathione (GSH). This reaction consumed MnO_2_ nanosheets, which acted as a catalyst for the oxidation of 3,3′,5,5′-tetramethylbenzidine (TMB) to a blue product (oxTMB). In the presence of Cu^2+^, the GSH was catalyzed to GSSG (oxidized glutathione), and the solution changed from colorless to deep blue. Under the optimum conditions, the absorption signal of the oxidized product (oxTMB) became proportional to Cu^2+^ concentration in the range from 10 to 300 nM with a detection limit of 6.9 nM. This detection system showed high specificity for Cu^2+^. Moreover, the system has been efficaciously implemented for Cu^2+^ detection in actual tap water samples. The layered-nanostructures of MnO_2_ nanosheets make it possess high chemical and thermal stability. TMB can be quickly oxidized within 10 min by the catalyzing of MnO_2_ nanosheets with high oxidase-like activity. There is no need of expensive reagents, additional H_2_O_2_ and complicated modification processes during the colorimetric assay. Therefore, the strategy primarily based on MnO_2_ nanosheets is promising for real-time, rapid and highly sensitive detection of Cu^2+^ under practical conditions.

## Introduction

Copper is an essential microelement for the human body and an important component of human proteins and enzymes. Lack of copper ions (Cu^2+^) will hinder the physiological activities of human body and easily cause various diseases (Scheiber et al., 2013; Chowdhury et al., 2018). On the other hand, copper is a heavy metal widely discovered in the environment, and excessive copper will produce severe toxic effects on humans, plants and microorganisms (Lee et al., 2016; Zhou et al., 2020). With the extensive utility of copper in industry and agriculture, it has become one of the main pollutants of social concern. Therefore, developing sensitive strategies to detect Cu^2+^ in environmental and biological samples is essential.

The detection techniques of Cu^2+^ have been greatly developed, such as atomic absorption spectroscopy (AAS) (Lima et al., 2012), inductively coupled plasma mass spectrometry (ICP-MS) (Dai et al., 2012; Khan et al., 2014), electrochemical techniques (Flavel et al., 2011; Zhu et al., 2017), fluorescence methods (Lan et al., 2010; Zhang et al., 2014; Zhang et al., 2020) and colorimetric assays (Ma et al., 2011; Weng et al., 2013; Le et al., 2014; Wang et al., 2017; Luo et al., 2020). Some of these methods suffer from the drawbacks of requirements for large instruments, special operators, complex sample pretreatment process, or poor reproducibility and reliability, which limits their on-side rapid detection and practical application (Du et al., 2014; Wang et al., 2019a). Among them, colorimetric analysis is concerned because of its simple and quick operation, low cost, visualization and no need for professional operators, which makes it widely applied.

Colorimetric assays are generally based on peroxidase or some nanomaterials as the mimic enzyme to initiate chromogenic reactions that produce colored compounds (Jangi et al., 2020; Wang et al., 2020; Xia et al., 2021). Many chromogenic reactions are carried out via catalyzing the peroxidase substrates, usually TMB or 2, 2′-azinobis (3-ethylbenzothiazoline-6-sulfonic acid) (ABTS) (Tang et al., 2016). These reactions generally need additional hydrogen peroxide as the oxidant, which complicates the operation and increase the background signal. Strict time-controlling is necessary to improve the signal to noise ratio. There is also some other special color reactions, such as the etching of gold nanorods showing various color changes (Lin et al., 2016), the pink-blue transition caused by the aggregation of gold nanoparticles (Deng et al., 2013), and other methods generating colored compounds (Yin et al., 2015). Several colorimetric assays based on the nanomaterials of gold, silver and platinum nanoparticle have been developed for sensitive detection of Cu^2+^ (Ma et al., 2011; Le et al., 2014; Wang et al., 2017). The scarcity, high cost and easily poisoned of these precious noble metals, have hindered their large-scale use in practical colorimetric system (Kuo et al., 2014; Asif et al., 2017b). As a result, the creation of more economical and environment-friendly materials for colorimetric detection of Cu^2+^ is of significant importance.

In current years, some non-precious metals oxides-based nanosheets and nanosphere has been synthesized for electrochemical detection of various pollutants, such as MnO_2_, CuO and graphene (Asif et al., 2017a; Asif et al., 2017b; Asif et al., 2018; Asif et al., 2019a; Aziz et al., 2019). MnO_2_ nanosheets (NSs) have obtained high-level interest as a brand new type of two-dimensional (2D) nanomaterial (Chen et al., 2019). MnO_2_ nanosheet is a kind of graphene-like nanomaterial, which can be used as a DNA nanocarrier to construct ratiometric fluorescence biosensors to detect miRNA and living cell imaging (Wang et al., 2019a). Due to its large particular sensing area, high chemical durability and inherent oxidative enzyme mimic activity, MnO_2_ NSs are widely utilized in biosensors. The strong oxidation ability of MnO_2_ NSs makes it great potential to constitute a colorimetric strategy for practical applications. Also, MnO_2_ has excellent ion exchange and redox capabilities that can be widely used in supercapacitors, batteries and catalysis. It has been identified as the most promising electrode material for electrochemical energy storage systems, depending on its high density, high purity and sufficient electrochemical activity (Guo et al., 2019). some MnO_2_ NSs based material has been synthesized for photoelectrochemical detection of Cu^2+^ (Hammami et al., 2021), electrochemiluminescence detection of glutathione (Gao et al., 2016), fluorescence detection of Fe^2+^ (Jiang et al., 2022), ascorbic acid (Xu et al., 2017), and glutathione (Wang et al., 2016), colorimetric detect of glutathione (Ge et al., 2019), acetylcholinesterase (Yan et al., 2017) and chlorothalonil (Sheng et al., 2020). Nevertheless, the colorimetric assays based on MnO_2_ NSs for Cu^2+^ detection are very rare.

Herein, based on the catalytic oxidation activity of MnO_2_ NSs, an easy, speedy and economic colorimetric assay is established for the sensitive determination of Cu^2+^. TMB can be quickly oxidized by MnO_2_ NSs to form blue oxTMB without the need for H_2_O_2_. After interaction with reduced glutathione (GSH), the MnO_2_ NSs are dissolved, which reduce the formation amount of blue oxTMB, so the mixture remains colorless. When Cu^2+^ ions are introduced into the reaction mixture, Cu^2+^ catalyzes the formation of GSSG (oxidized glutathione) from GSH and thus inhibits the decomposition of MnO_2_ NSs. As a result, the color of the mixture changes from colorless to blue. According to these results, a simple colorimetric method for Cu^2+^ was established. Moreover, this method has high sensitivity and selectivity for the determination of Cu^2+^. The applicability of the assay in practical samples was also investigated.

## Methods and Materials

### Materials and Reagents

Tetramethylammonium hydroxide (TMAOH), cysteine (Cys), ascorbic acid (AA), uric acid (UA) were purchased from Aladdin Reagent Co., Ltd (Shanghai, China). Bovine serum albumin (BSA) and l-glutathione (GSH) were received from Sigma-Aldrich (St. Louis, Missouri, United States ). Manganese chloride tetrahydrate (MnCl_2_ **.**4H_2_O), hydrogen peroxide (H_2_O_2_, 30 wt%), sodium hydroxide (NaOH), copper sulfate (CuSO_4_ **.**5H_2_O), acetic acid (HAc), and ethanol were obtained from Sinopharm Chemical Reagent Co. (Shanghai, China). TMB substrate solution was obtained from Beyotime Biotechnology (Shanghai, China). All of the reagents used were analytical grades and were used directly (without further treatment). All the solutions were prepared from ultrapure water produced by a Milli-Q system.

### Apparatus and Instrumentation

UV-Vis absorption spectra had been measured on the Cary-300 UV-Vis spectrophotometer (Agilent, United States ). X-ray photoelectron spectroscopy (XPS) spectra were measured on the ESCALAB MKⅡX-ray photoelectron spectrometer (Thermo Fisher Scientific, United States ). Morphology of MnO_2_ NSs was analyzed by Nova NanoSEM 230 field-emission scanning electron microscopy (FEI, United States ). Transmission electron microscope (TEM) photographs of MnO_2_ NSs were obtained on the JEM-2100 transmission electron microscope (JEOL, Japan). The level of metal ions in practical water samples was evaluated by an X SERIES II inductively coupled plasma mass spectroscopy (ICP-MS) (Thermo Fisher Scientific, United States ).

### Synthesis of MnO_2_ NSs

MnO_2_ NSs were prepared according to the previous literature (Liu et al., 2017). Briefly, the mixture composed of 4.4 mL (25 wt%) TMAOH, 2 mL (3 wt%) H_2_O_2_ and 15 mL ultrapure water was quickly added into 10 mL MnCl_2_.4H_2_O solution (0.3 M) within 15 s. Then, the resulting dark brown suspension was stirred vigorously overnight at room temperature. After that, the prepared bulk manganese dioxide was centrifuged at 8,000 rpm for 10 min and washed three times with a large amount of distilled water and alcohol. The washed precipitate and 20 mg of BSA were dispersed in 20 mL ultrapure water and then treated with ultrasound for 10 h. The suspension was then centrifuged at 4,000 rpm for 15 min to remove the undispersed pellet. The supernatant was placed in the dark at 4°C for further use.

### Colorimetric Detection of Cu^2+^

Copper sulfate was dissolved in ultrapure water to prepare various concentrations of Cu^2+^ solutions. At first, 14 μL of 0.5 mM GSH solution was mixed with 40 μL different concentrations of Cu^2+^ solutions and reacted for 5 min. Then, 15 μL MnO_2_ NSs (1.3 mg/mL) and 200 μL citric acid buffer (0.01 M, pH = 5.0) were added to the mixture and maintained 10 min at 45°C to ensure complete reaction. Subsequently, 100 μL of TMB substrate solution was introduced into each tube for another 10 min. Finally, the absorbance of the reaction solution in the wavelength range of 500–800 nm was determined. All reactions were carried out at 45°C.

### Selectivity Investigation of Cu^2+^ Assay

In order to study whether other ions might interfere with the detection of Cu^2+^, some other metal ions were applied to the system with ten times concentrations of that of Cu^2+^. These ions including Fe^3+^, Ni^2+^, Ca^2+^, Mg^2+^, K^+^, Zn^2+^, Mn^2+^, Pb^2+^, Na^+^, Hg^2+^, and Ag^+^. 1.0 μM Cu^2+^ was used for the test and the concentration of other metal ions was 10 μM.

### Detection of Cu^2+^ in Actual Samples

To investigate the application ability of the colorimetric method in the practical samples, the collected tap water and waste water were naturally settled overnight for experiments. The supernatant obtained was treated with the 0.45 μm filtration membrane to take away particulate impurity. Then different concentrations of Cu^2+^ were introduced to the filtrate for further detection. The procedure described above was used for Cu^2+^ detection in the tap water and waste water samples.

## Results and Discussion

### The Principle of Colorimetric Determination of Cu^2+^

**SCHEME 1:**
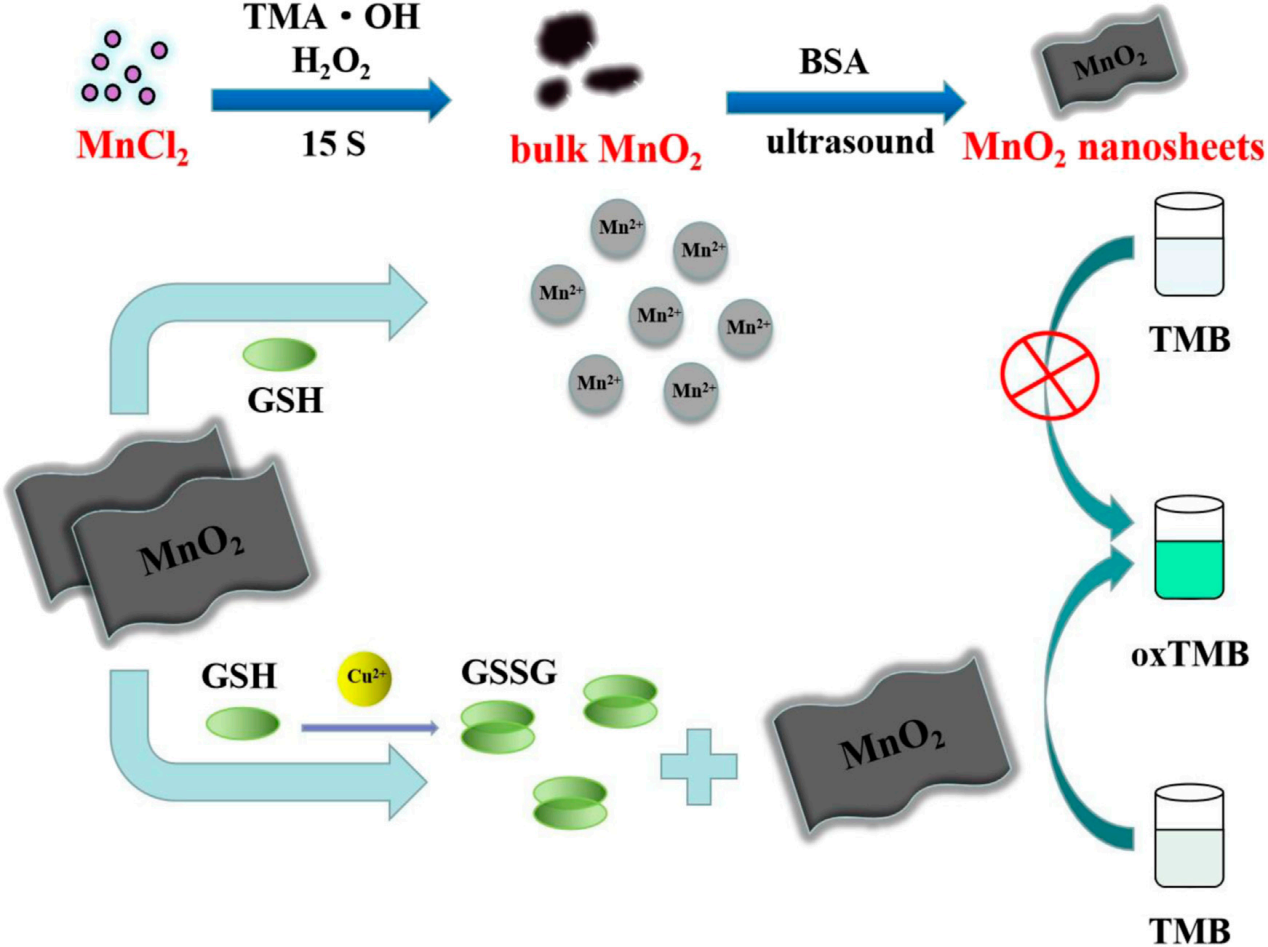
Schematic diagram of colorimetric determination of Cu^2+^ based on MnO_2_ NSs.

The strategy of Cu^2+^ colorimetric assay based on MnO_2_ NSs is described in Scheme 1. Due to its intrinsic oxidase-like activity, the synthesized MnO_2_ NSs can catalyze the formation of blue oxidation product (oxTMB) from colorless substrate TMB, which possesses an absorption peak at 650 nm. No additional H_2_O_2_ is added during this process. As an antioxidant, GSH can react with MnO_2_ NSs in the way shown in Eq. 1, resulting in the reduction and decomposition of MnO_2_ NSs. The released Mn^2+^ loses the catalytic ability, thus the chromogenic reaction of TMB is inhibited. In the presence of Cu^2+^, Cu^2+^ can catalyze GSH to GSSG (Tang et al., 2016), resulting in less decomposition of MnO_2_ NSs and more generation of oxTMB. As a result, the color of the solution turns blue. Cu^2+^ concentration is linearly related to the absorption value of TMB oxidation products. Thus, a sensitive “turn on” Cu^2+^ detection scheme is established.

$$\mathrm{Mn}O_{2}+2\mathrm{GSH}+2H^{+}\to Mn^{2+}+\mathrm{GSSG}+2H_{2}O$$ (1)

### Feasibility of the Cu^2+^ Colorimetric Assay

**FIGURE 1:**
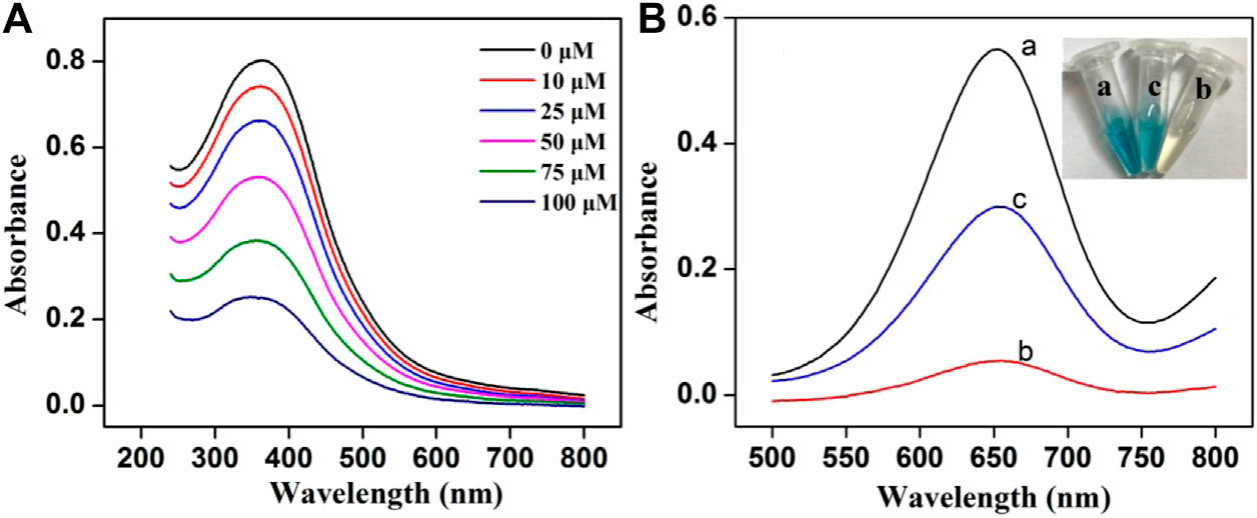
**(A)** The UV-vis absorption spectra of MnO_2_ NSs (162.5 μg/mL) with different concentrations of GSH **(B)** Absorption spectra of the system under different conditions: (a) MnO_2_ + TMB, (b) MnO_2_ + GSH + TMB and (c) MnO_2_ + Cu^2+^ + GSH + TMB (Inset: the photograph of the solution color).

Some control experiments were performed to confirm the feasibility of the colorimetric assay. As shown in Figure 1A, the characteristic peak (380 nm) of MnO_2_ NSs gradually decreased with the increasing GSH concentration, indicating that MnO_2_ NSs have been decomposed by GSH. Without GSH, the TMB oxidation process can be promoted by MnO_2_ NSs, generating colored solution and significant absorption signal (Figure 1B, curve a). After interaction with GSH, the MnO_2_ NSs was decomposed, and TMB oxidization was inhibited, which resulted in colorless solution and a great decrease of absorption signal (Figure 1B, curve b). Through the catalysis of Cu^2+^, GSH was oxidized to GSSG, which inhibited MnO_2_ NSs dissociation. Thus the absorption signal was enhanced (Figure 1B, curve c). According to these results, we concluded that Cu^2+^ could be quantified by measuring the absorption values of TMB oxidation products at 650 nm.

### Characterization of MnO_2_ NSs

**FIGURE 2:**
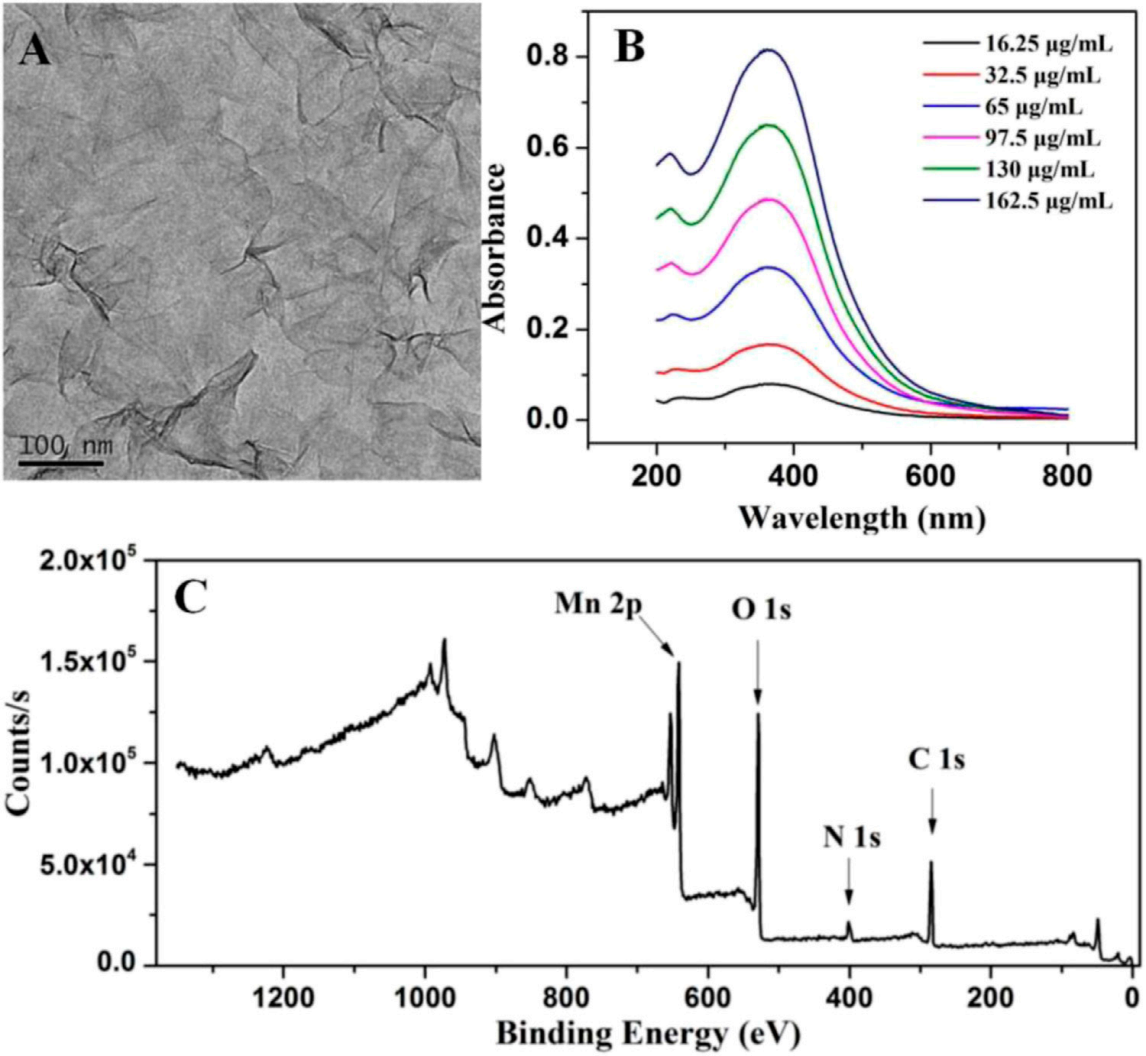
**(A)** TEM photograph of MnO_2_ NSs **(B)** Absorption spectra of different concentrations of MnO_2_ NSs **(C)** XPS characterization of MnO_2_ NSs.

After interaction with TMAOH, Mn^2+^ was oxidized to Mn^4+^ by H_2_O_2_ to form bulk MnO_2_. By treating it with BSA under ultrasonication, MnO_2_ NSs with good dispersibility, biocompatibility and high peroxidase activity were obtained, as previously described (Liu et al., 2012). The morphology of MnO_2_ NSs was analyzed by scanning electron microscopy (SEM). As displayed in Supplementary Figure S1, the obtained MnO_2_ NSs presented an obvious sheet-like morphology and well dispersed in water. As the TEM image (Figure 2A) shown, the obtained MnO_2_ NSs exhibit characteristic 2-D layered architecture with partial folds/wrinkles, which contribute to the large specific surface area and good dispersibility.

The UV-Vis absorption spectra in Figure 2B shows that MnO_2_ NSs have a wide absorption spectra in the 250–500 nm range with a maximum absorption band at 380 nm, which can be ascribed to a d-d jump of Mn^4+^. This is in agreement with the optical properties of MnO_2_ NSs reported previously (Liu et al., 2012). Furthermore, with the increase of MnO_2_ NSs, the absorption intensity at 380 nm increased.

**FIGURE 3:**
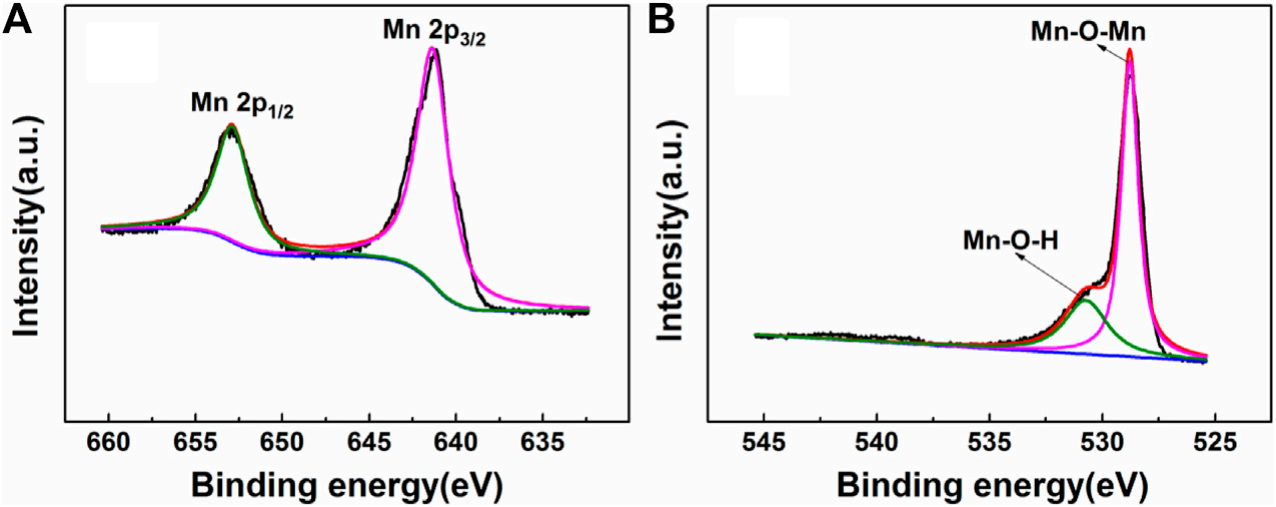
The XPS spectra of **(A)** Mn 2p and **(B)** O 1 s.

Chemical and elemental compositions of MnO_2_ NSs were analyzed by XPS. The wide scan spectrum results are represented in Figure 2C. Four main peaks centered at 647.2, 528.7, 401.6 and 284.7 eV can be ascribed to Mn 2p, O 1s, N 1s and C 1s (Liu et al., 2012; Asif et al., 2019b). Figure 3 shows the high-resolution XPS spectra of Mn 2p and O 1s. From Figure 3A, two strong characteristic peaks located at 652.9 and 641.2 eV emerge in the Mn 2p_1/2_ and Mn 2p_3/2_ core-level spectra, corresponding to the presence of Mn^4+^ moieties (Iftikhar et al., 2021). In Figure 3B, the peak at 528.7 eV is caused by O 1s. The O 1 s spectrum contains two sub energy states in Figure 3B and these peaks can be assigned to Mn–O–Mn and Mn–O–H (Liu et al., 2012; Asif et al., 2022; Aziz et al., 2022).This indicate that the acquisition of pure MnO_2_ and the oxidation valence of Mn is +4 (Saha and Pal, 2014). These results indicate the successful synthesis of MnO_2_ NSs.

### Optimization of the Experimental Conditions

In order to improve the sensitivity of the Cu^2+^ detection system, we further optimized some important experimental conditions, including pH, reaction temperature, the dosage of TMB substrate solution and GSH concentration. At first, the optimal pH of the reaction system was studied. HAc-NaAc buffer solutions with different pH were added to the reaction system. Supplementary Figure S2 shows the absorption values at different pH in the presence and absence of Cu^2+^. In the presence of 250 nM Cu^2+^, the absorption increment increases sharply in the pH range of 3.5–5.0. While the pH reaches 5.5, the increment of absorption decreases instead. As continues to raise the pH, the absorption increment returns to a slow upward trend. It is determined that the absorption increment is maximal when the pH value is 5.0. Therefore, the pH was selected at 5.0 for further test.

We also studied the influence of temperature on Cu^2+^ sensing systems. The absorption values at different temperatures in the presence and absence of Cu^2+^ are shown in Supplementary Figure S3. In the range of 25–45°C, the increase of temperature is beneficial to the reaction, and the absorbance difference of the final solution presents a steady rise trend. When the temperature reaches 45°C, the absorbance difference reached the maximum. Once the temperature is above 45°C, the temperature has a negative impact on this system. In order to obtain good experimental results, the colorimetric Cu^2+^ detection was carried out at the temperature of 45°C.

Subsequently, it was found that the amount of TMB substrate solution was related to the absorption intensity of the reaction solution at 650 nm. As Supplementary Figure S4 shown, the absorption value increases gradually with the increase of TMB substrate solution and remains stable when the amount of TMB is more than 100 μL. Therefore, the dosage of TMB substrate solution was selected as 100 μL.

The concentration of GSH used to decompose MnO_2_ NSs is critical. In order to achieve higher detection sensitivity, it was also optimized. As shown in Supplementary Figure S5A, the absorption value of the solution gradually decreases with the increase of GSH concentration in the reaction system. The solution color also changes from blue to colorless. These results indicated that MnO_2_ NSs were decomposed into Mn^2+^ by GSH, which inhibited the production of blue oxTMB. Meanwhile, there was a good linear relationship between the absorbance and the concentration of GSH from 0 to 18.75 μM (Supplementary Figure S5B). Considering the sensitivity and practical conditions of the system, the colorimetric assay was performed by selecting 17.5 μM GSH.

### Sensitivity, Reproducibility and Stability Investigation of Cu^2+^ Assay

**FIGURE 4:**
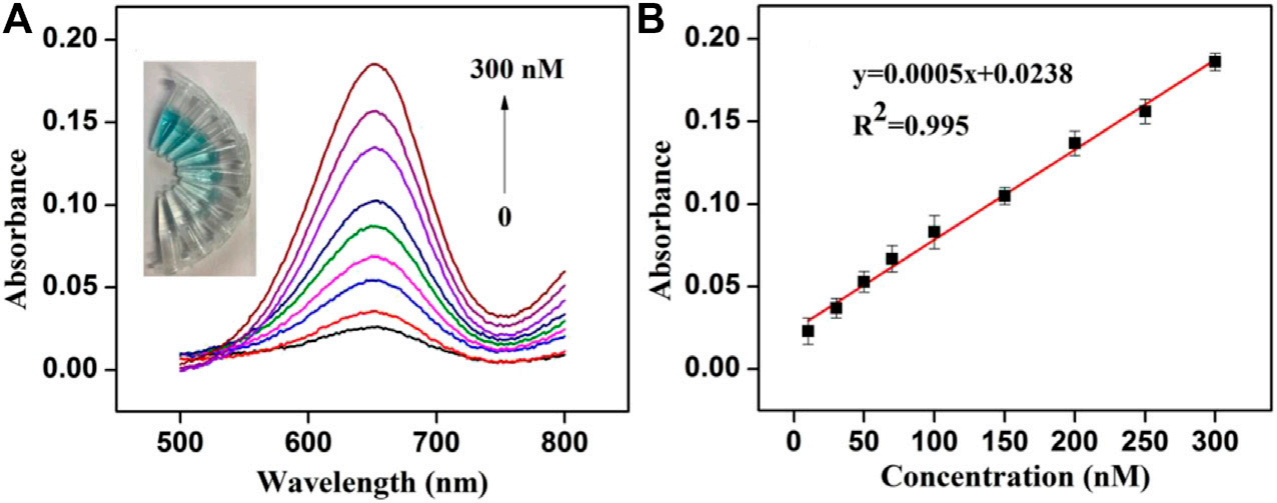
**(A)** Absorption spectra obtained for colorimetric detection of Cu^2+^ (Inset: the photograph of the color change of the solution) **(B)** Linear relationship between Cu^2+^concentration (10–300 nM) and the absorption value.

The sensitivity of the colorimetric assay was verified by adding different concentrations of Cu^2+^ to the solution at the optimal conditions. The recorded UV-vis absorption spectra of the system and the calibration curve established by absorption values of different concentrations of Cu^2+^ at 650 nm are presented in Figure 4. The results showed that with the increase of Cu^2+^ concentration, absorption value exhibited a rising trend and the color of the solution became deeper (Figure 4A). The absorption value has a good linear relationship with Cu^2+^ concentration from 10 to 300 nM, and the correlation coefficient is 0.997 (Figure 4B). Cu^2+^ detection limit (LOD) was estimated to about 6.9 nM (3σ). Because of the hazardous effects of high levels of Cu^2+^, the US Environmental Protection Agency stipulates that the maximum level of Cu^2+^ allowed in drinking water was about 20 μM (U.S.E.P. Agency, 2012). Thus, this colorimetric assay is suitable for the detection of low concentration Cu^2+^ in the environment.

Compared with other reported methods in Table 1, this strategy could achieve a lower detection limit and a wider detection range. Besides, the characteristic of the novel colorimetric assay, such as simple operation (no additional H_2_O_2_ was required), low toxicity, and low cost (without the need of precious metal or nucleic acid), makes it great potential in high sensitivity and rapid test of Cu^2+^ in drinking water and environment.

**TABLE 1:** The list comparison of this work with other previously published works.

Method	Material	Analytical ranges	LOD	Ref
Fluorescence	Upconversion nanoparticles	0.125–3.125 µM	100 nM	Su et al. (2021)
Fluorescence	Ag_2_S quantum dots	25 nM∼10 μM	27.6 nM	Jiang et al. (2019)
Fluorescence	HP-GO^a^	0–3.93 μM	54 nM	Awad et al. (2020)
Fluorescence	CdTe quantum dot	0–1,000 nM	1.45 nM	Ye et al. (2020)
Colorimetric	MMoO_4_ ^b^	0.1–24 μM	24 nM	Luo et al. (2020)
Colorimetric	Au@Pt nanocatalysts	20–300 nM	3.7 nM	Wang et al. (2017)
Colorimetric	ZnO-Co_3_O_4_ nanocages protein-based nanoprobe	2–100 nM	1.08 nM	Lv et al. (2021)
Colorimetric	protein-based nanoprobe	0–8 μM	160 nM	Hu et al. (2021)
Electrochemistry	Au Nanoparticles	2.5–25 μg/L	0.9 μg/L	Wang et al. (2019b)
Electrochemistry	IIP-film^c^	0.95–244 nM	2.7 nM	Di Masi et al. (2020)
Colorimetric	GSH/MnO_2_ NSs	10–300 nM	6.9 nM	This work

a Hematoporphyrin modified graphene oxide.

b M = co, Ni.

c Ion imprinted polymeric film.

The reproducibility of the colorimetric sensor was evaluated by intra-assay. Five parallel measurements have been performed to detect 100 nM Cu^2+^ at the same conditions. The obtained relative standard deviation (RSD) was calculated to be 2.9%. The long-term stability of the colorimetric sensor was also investigated. The MnO_2_ NSs were stored in dark under 4 °C when it is not used. After stored for more than 1 month, the Cu^2+^ induced absorption response could retain 96% of its original value. These results indicate that the colorimetric sensor shows good repeatability and stability.

### Interference Study

**FIGURE 5:**
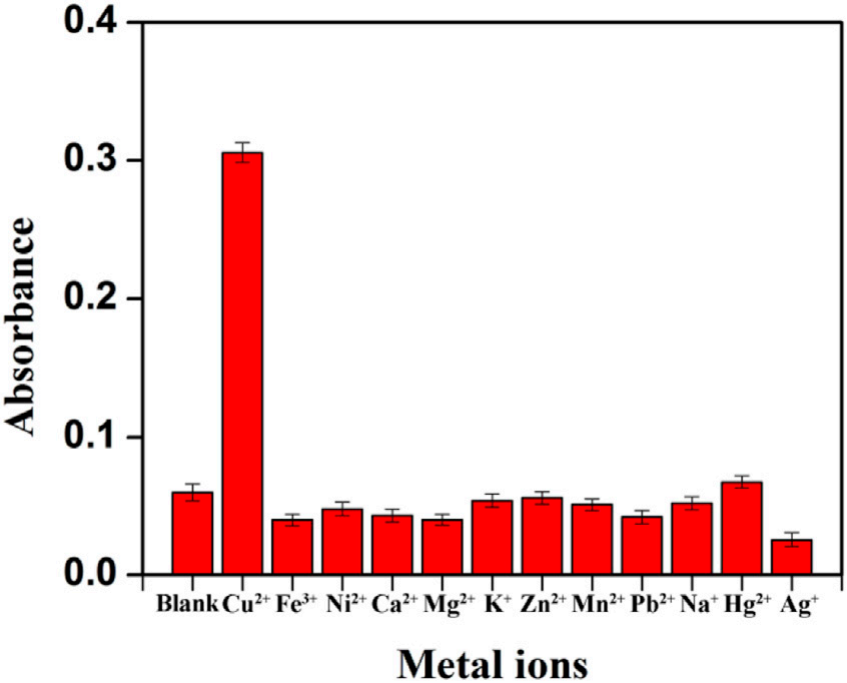
Study on the selectivity of the established Cu^2+^ assay after the addition of masking agents (The concentration of Cu^2+^ was 1.0 μM, other metal ions concentration was ten-fold of Cu^2+^).

To investigate the specificity of colorimetric determination of Cu^2+^, series interference experiments were also carried out. Various metal ions including Fe^3+^, Ni^2+^, Ca^2+^, Mg^2+^, K^+^, Zn^2+^, Mn^2+^, Pb^2+^, Na^+^, Hg^2+^, Ag^+^ and common compounds with redox properties were studied. Under the same conditions, 1.0 μM Cu^2+^ or other metal ions with ten times the concentration of Cu^2+^ were added to the system to collect the absorption signal. Although Ag^+^ and Hg^2+^ could give rise to measurable absorbance signals (Supplementary Figure S6) in the concentration of 10 μM, the interference is small when they are at the same concentration as Cu^2+^ (1.0 μM) (Supplementary Figure S7), and they will not significantly affect the detection of Cu^2+^ at this concentration in the environmental system. In addition, KBr (1.25 mM) and KCl (12.5 mM) were used as masking agents to eliminate the interference of Hg^2+^ and Ag^+^. It could be seen in Figure 5 that with the coexistence of KBr and KCl, the colorimetric assay showed high specificity for Cu^2+^. At the same time, other metal ions with concentration ten times of Cu^2+^ made no difference in the sensitivity of Cu^2+^ analysis. Common redox substances, such as 100 μM of Cys, AA, UA and 1.0 mg/mL of BSA had no significant effect on the detection of Cu^2+^ (Supplementary Figure S7). It is worth noting that the selectivity can be observed with the naked eye. Hence, the specific detection of Cu^2+^ can be achieved by the established colorimetric assay.

### Detection of Cu^2+^ in Real Samples

To verify the applicability of this method in practical samples and complex sample matrix, Cu^2+^ spiked tap water (collected from the laboratory) and waste water (collected from a point source suspected to discharge metal ions by Longyan Rare Earths LTD.) were prepared for the test. The practical samples were settled overnight at room temperature, and then the impurities were removed by filtration with a 0.45 μm filter membrane. For comparison, ICP-MS was also used to evaluate the Cu^2+^ level in these samples. The initial concentration of Cu^2+^ detected by ICP-MS in tap water and waste water is 3.5 and 235.2 nM, respectively. The analytical performances of the proposed colorimetric assay for the detection of Cu^2+^ from waste water is in good agreement with the results obtained by ICP-MS with RSD value of 1.74% (Supplementary Table S1). This result indicated the good accuracy of the proposed colorimetric assay for the analysis of Cu^2+^ in real samples. After dilution, series of Cu^2+^ with different concentrations were added into the pretreated water samples and detected by this colorimetric method. The detection results are listed in Supplementary Table S1. The recovery of standard Cu^2+^ spiked samples is between 95.2 and 105.7%, with RSD< 3.24% (n = 3). The results illustrate that this colorimetric method is promising for applying efficient quantitative detection of Cu^2+^ in actual samples with high sensitivity.

## Conclusion

To summarize, we developed a novel colorimetric method to detect Cu^2+^ rapidly and effectively. The basic principle of the method is that Cu^2+^ can catalyze GSH to GSSG, thus inhibiting the decomposition of the single-layer MnO_2_ NSs into Mn^2+^ by GSH to achieve Cu^2+^ detection. Under the optimal experimental conditions, the colorimetric system exhibits highly sensitive, broad linear range, low detection limit and rapid analysis. In addition, the experimental results showed that this method is expected to be used for the determination of Cu^2+^ in practical conditions. Compared to natural enzymes, the layered nanostructures of 2D MnO_2_ NSs makes themselves possess higher chemical and thermal stability. It should be noted that the TMB can be quickly oxidized by MnO_2_ NSs with high oxidase-like activity within 10 min. Importantly, the colorimetric assay does not require any expensive reagents, additional H_2_O_2_ or complicated modification processes and can complete the detection under mild conditions in a short time, which is expected to be applied to the detection of Cu^2+^ in other practical situations. It is worth mentioning that combining with Cu-contained nanomaterials, this colorimetric method can provide a novel general sensing strategy for the indirect detection of a variety of analytes.

## Data Availability

The original contributions presented in the study are included in the article/Supplementary Material, further inquiries can be directed to the corresponding authors.
