# Supplementary material for: A Simple Colorimetric Assay for Sensitive Cu2+ Detection Based on the Glutathione-Mediated Etching of MnO2 Nanosheets
Source: Front Chem. 2021 Dec 24;9:812503. doi: 10.3389/fchem.2021.812503 (PMC8739952; doi:10.3389/fchem.2021.812503)
Supplement: Supplementary file 1 [file DataSheet1.docx]

Supplementary Material


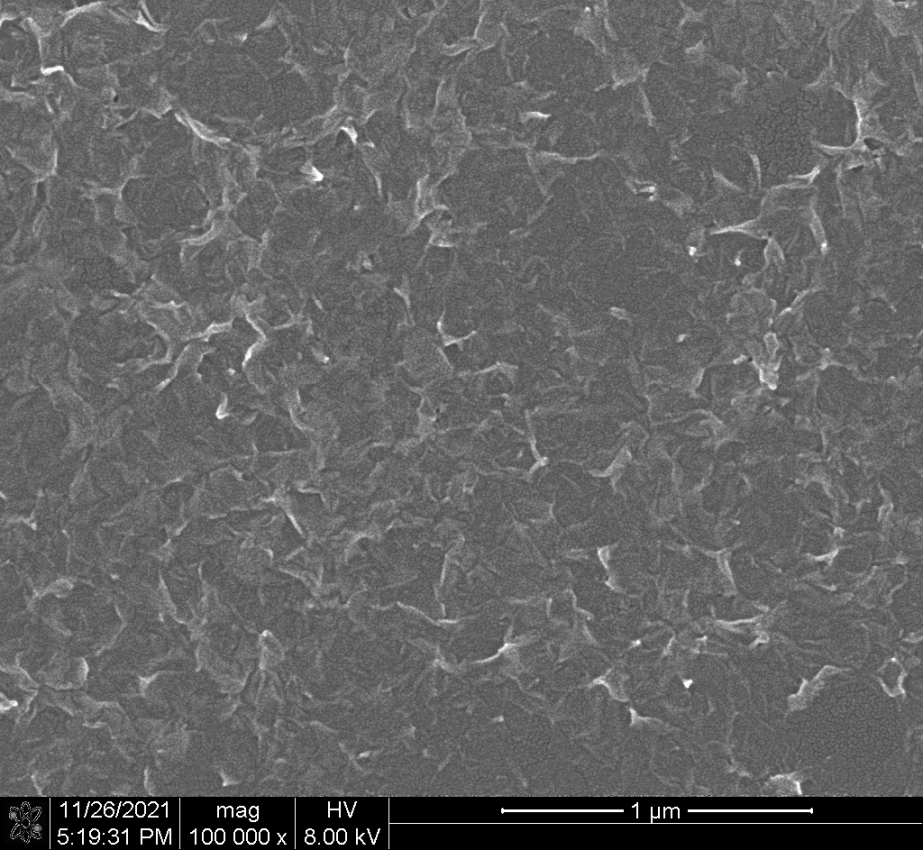


**Supplementary Figure S1.** SEM image of MnO_2_ NSs.


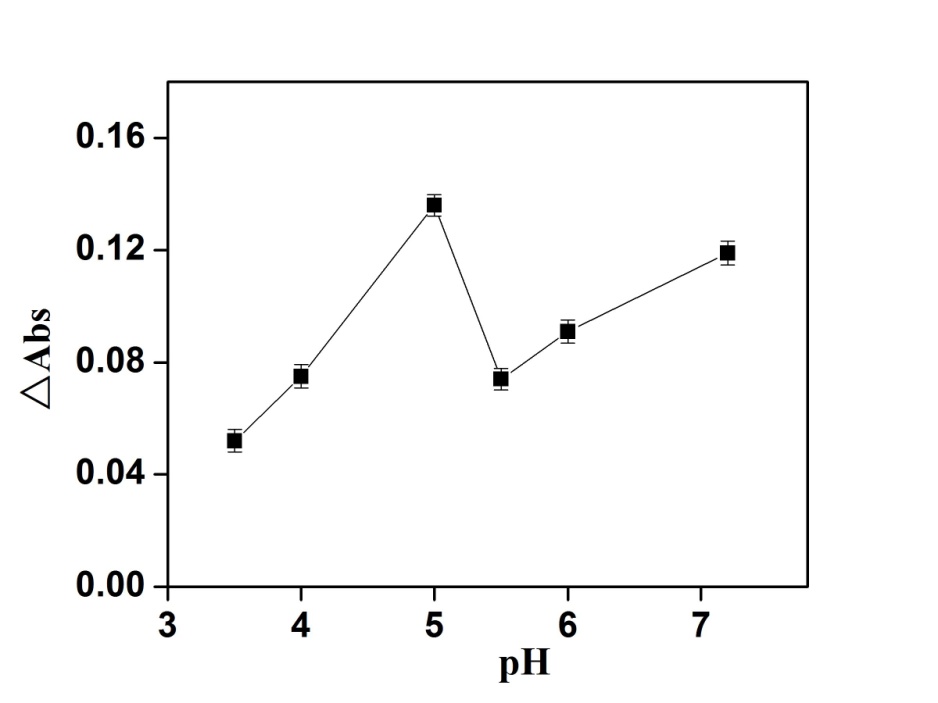


**Supplementary Figure S2.** Absorption increment values (∆Abs, in the presence and absence of copper ions) at different pH.


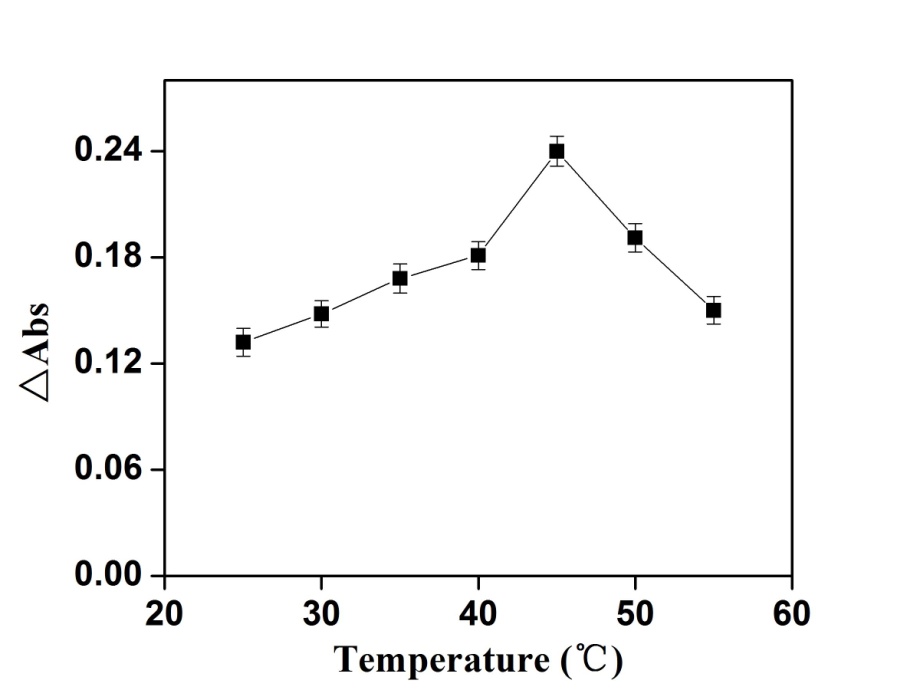


**Supplementary Figure S3.** Absorption increment values (∆Abs, in the presence and absence of copper ions) at different temperature.


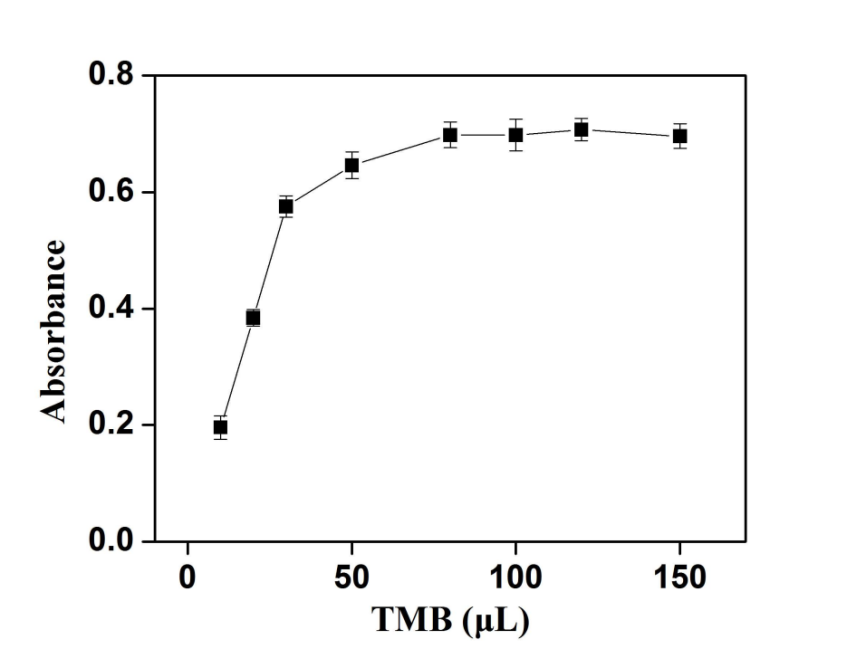


**Supplementary Figure S4.** Optimization of the amount of TMB substrate solution.

**
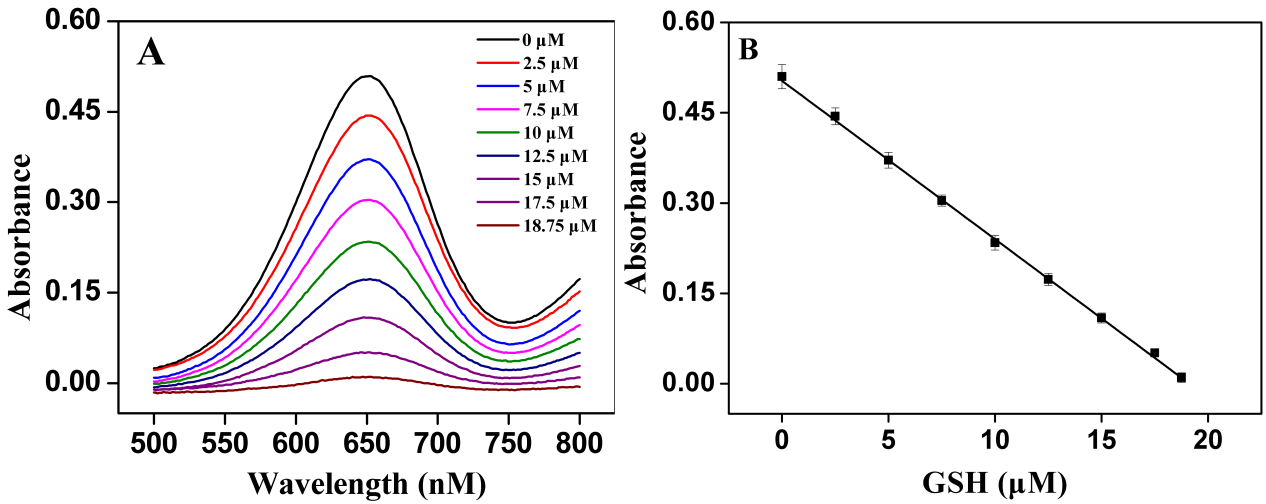
**

**Supplementary Figure S5.** **(A)** The absorption spectra of reaction solution containing MnO_2_ NSs (48.75 μg/mL), TMB (100 μL) and GSH with different concentration; **(B)** Absorption values at 650 nm of solution with different concentration of GSH.


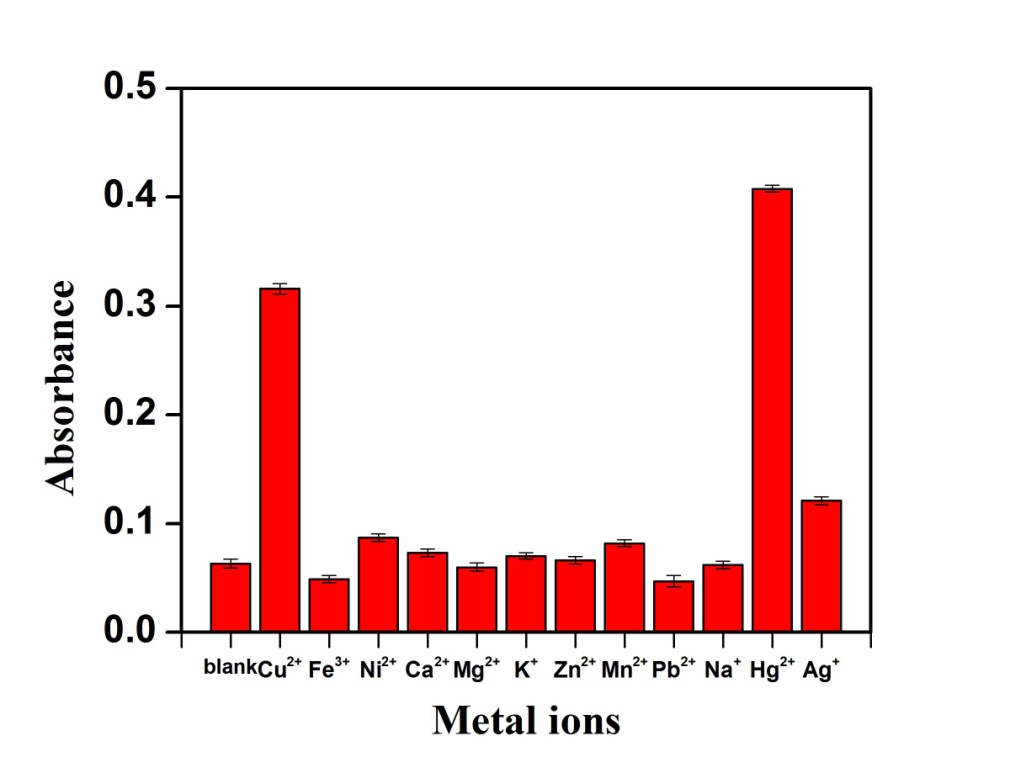


**Supplementary Figure S6.** The absorption value of the solution with different metal ions in the absence of masking agent (The concentration of Cu^2+^ and other metal ions was 1.0 μM and 10 μM, respectively).





**Supplementary Figure S7.** Selectivity of the established sensor for Cu^2+^ detection (The concentration of Cu^2+^, Hg^2+^ and Ag^+^ was 1.0 μM and other metal ions was 10 μΜ, the concentration of Cys, AA, UA was 100 μM and BSA was 1.0 mg/mL).

**Supplementary Table S1** Results of the Cu^2+^ colorimetric assay performed in tap water.

| Sample | ICP-MS/nM | Add/nM | Found/nM | Recovery/% | RSD/% |
| --- | --- | --- | --- | --- | --- |
| Tap water  Waste water | 3.5  235.2 | 0  50.0  100.0  200.0  0  30.0  150.0  250.0 | ND  47.6  102.2  208.9  243.5  31.6  148.7  264.3 | -  95.2  102.2  104.5  -  105.3  99.1  105.7 | -  1.28  2.13  0.65  1.74  3.24  2.48  1.96 |
